# Clinical Outcomes Associated with the Use of a Family-Based Digital Support Program in Patients with Pharmacologic Treatment for Obesity

**DOI:** 10.3390/jcm15010222

**Published:** 2025-12-27

**Authors:** Antonio de Arriba Muñoz, Oscar Eduardo Rodríguez-Montes, Ana Rocío Conde-Moro, María Teresa Garcia Castellanos, José Andrés Martínez García, Luis Fernández-Luque

**Affiliations:** 1 Pediatric Endocrinology Unit, Miguel Servet Children’s University Hospital, 50009 Zaragoza, Spain; tgcastellanos24@gmail.com (M.T.G.C.); jamartines@iisaragon.es (J.A.M.G.); 2 Aragon Health Research Institute, 50009 Zaragoza, Spain; 3 Adhera Health SL, 41092 Sevilla, Spain; orodriguez@adherahealth.com (O.E.R.-M.); aconde@adherahealth.com (A.R.C.-M.); luis@adherahealth.com (L.F.-L.)

**Keywords:** DHIs, childhood obesity, GLP-1 treatment, pediatric obesity interventions, mHealth, family-centered care, pediatric endocrinology

## Abstract

**Background/Objectives:** The Adhera^®^ Caring Digital Program (ACDP) is a digitally delivered intervention aimed at enhancing the mental and physical well-being of family caregivers of children with chronic conditions. Tailored for the context of childhood obesity, ACDP leverages the Adhera AI Precision Digital Companion to support caregivers in promoting effective treatment adherence and healthy behaviors at home. While children in this study received GLP-1 receptor agonist treatment as part of their obesity management, the ACDP was designed to empower caregivers through educational, behavioral, and emotional support tools. The aim of this study was to determine the relationship between engagement with the ACDP as a DHI and clinical outcomes in participants receiving GLP-1 receptor agonist treatment for obesity. **Methods:** This study analyzed data from approximately 40 pediatric patients receiving GLP-1-based pharmacological treatment for obesity and their caregivers enrolled in the ACDP. Caregiver engagement was assessed through a composite score derived from digital activity metrics and classified as low, medium, or high. Children’s clinical parameters (BMI, weight, fat mass %, muscle mass %, and waist circumference) were collected at baseline and Day 150. Biometric, physical activity, and sleep data were also collected through a wearable device, enabling continuous and objective monitoring of participants’ physiological and behavioral patterns in real-world conditions. Statistical analyses included paired comparisons, Pearson correlations, and group comparisons by engagement level. **Results:** Data from 40 pediatric participants and their caregivers were analyzed over 150 days. Observed changes over the time in children showed a reduction in waist circumference (−6.0%, *p* = 0.0056) and a modest decrease in BMI. Higher levels of caregiver engagement with the Adhera^®^ Caring Digital Program correlated with reduction in body fat (ΔFat% r ≈ 0.5, *p* = 0.091) representing the strongest correlation observed in the study, albeit not reaching statistical significance. From baseline to Day 150, significant improvements were observed across all clinical outcomes, including reductions in BMI (−4.51 kg/m^2^), body weight (−11.42 kg), body fat percentage (−5.63%), and waist circumference (−8.69 cm), alongside an increase in muscle mass (+4.47%) (all *p* < 0.0001). **Conclusions:** At the mid-point of the intervention, combined GLP-1 pharmacotherapy and the Adhera^®^ Caring Digital Program led to meaningful improvements in BMI, adiposity, and central obesity. While caregiver engagement was not significantly associated with short-term outcomes, observed trends suggest that digital support may provide complementary benefits to pharmacological treatment, warranting further evaluation at Day 300.

## 1. Introduction

Childhood overweight and obesity represent a growing global public health concern. Their prevalence among children and adolescents aged 5 to 19 years has increased by 6.3%, rising from 1.9% in 1990 to 8.2% in 2022 [1]. The data reveal a rapid increase in overweight and obesity across all continents, with a high prevalence among children, particularly those approaching adolescence [2,3]. It is estimated that, worldwide, one in five children or adolescents has excess body weight—8.5% among children and 14.8% among adolescents—with various risk factors, including genetic, dietary, and environmental determinants, that are significantly associated with this condition [4].

In Spain, according to the ALADINO 2023 study, the prevalence of obesity reached 15.9% among children, with higher values in boys (18.2%) than in girls (13.5%) [5].

For affected children, this may lead to a range of short-term physical, social, and emotional difficulties—such as elevated blood pressure, early signs of cardiovascular disease, diabetes, and musculoskeletal complications—along with psychological distress and mental health conditions that are often intensified by social stigma [2]. Therefore, based on clinical practice guidelines, pharmacological treatments have been employed to address obesity in children, thereby reducing the risk of associated health complications. However, the decision to prescribe should be based on the individual case, considering the risks and benefits of the therapeutic option, and must be accompanied by lifestyle modifications and health behavior interventions [6].

According to these recommendations, various studies have used an approach that combines pharmacological treatment with non-pharmacological interventions involving lifestyle, diet, and exercise modifications [7,8,9], with better outcomes in clinical indicators than treatment relying on pharmacotherapy alone.

In the management of adolescents, pharmacotherapy is used as an indicated treatment in cases of persistent moderate-to-severe obesity, particularly when cardiometabolic risk justifies treatment, always within specialized care (pediatric endocrinology) or when lifestyle changes do not result in weight loss or reductions in BMI [7]; for this reason, a combined approach incorporating both pharmacological and non-pharmacological elements may be further strengthened through the use of digital interventions, which have demonstrated promising results in enhancing the adoption of healthy lifestyle behaviors among adolescents with obesity [9].

This article presents the preliminary results of a digital intervention corresponding to the evaluation period between Day 1 and Day 150. The aim was to explore which clinical variables showed significant changes following the combined use of pharmacological treatment and the digital intervention, which provided tailored recommendations on lifestyle modification, healthy habits, and parental support for families of children with obesity. Additionally, we examined the user experience with the ACDP in terms of usability and engagement levels to determine whether these variables exhibit meaningful intercorrelations.

## 2. Materials and Methods

### 2.1. Study Design

This is an observational, prospective, single-arm pre-post study aimed at evaluating the impact of a combined treatment for childhood obesity, which includes the ACDP^®^ digital intervention and pharmacological treatment. The study followed participants over a 10-month active intervention period, with an additional 2-month follow-up phase. The design and reporting of this study adhered to the Strengthening the Reporting of Observational Studies in Epidemiology (STROBE) and CONSORT extension guidelines for digital health interventions. The study was registered in the Protocol Registration and Submission of Study Documents and Results (PRS) system (ClinicalTrials.gov) under registration number NCT06520787 on 22 November 2024.

### 2.2. Settings and Participants

Participants were recruited from the Pediatric Endocrinology Unit at Miguel Servet Children’s University Hospital, a public tertiary care center serving the region of Aragón (Spain). Eligible families were identified through electronic medical records and invited to participate during routine outpatient visits. All measurements were obtained during routine pediatric endocrinology consultations using standardized clinical procedures. Paired *t*-test analyses were conducted to examine within-participant changes across this period.

### 2.3. Inclusion Criteria

- Caregivers of children aged 13–18 years diagnosed with obesity (BMI ≥ 95th percentile for age and sex, according to AEMPS criteria).
- Children under pharmacological treatment for obesity with semaglutide.
- Families willing to use the *Adhera^®^ Caring Digital Program* for 10 months and synchronize wearable devices throughout the study.
- Fluency in Spanish and access to a compatible smartphone (Android/iOS).

### 2.4. Exclusion Criteria

- Secondary causes of obesity (genetic, hypothalamic, or endocrine).
- Severe comorbidities or inability to comply with the digital intervention.
- Families not fluent in Spanish.

### 2.5. Sample Size

A total of 40 families were recruited based on feasibility estimates and prior pilot studies in similar pediatric populations.

### 2.6. Intervention

The combined treatment consisted of

1. Pharmacological therapy, prescribed according to clinical indication and current AEMPS/EMA-approved protocols (GLP-1 receptor agonists, metformin, or orlistat).
2. Digital intervention via ACDP^®^, a mobile-based precision health program developed by Adhera Health SL. The program integrates AI-driven personalization based on cognitive-behavioral principles, educational modules, motivational messaging, and progress feedback.

Caregivers were onboarded through a 60 min training session covering app installation, device synchronization, and completion of baseline assessments. Clinical data were reported by the pediatric endocrinology team at Miguel Servet University Children’s Hospital according to the scheduled follow-up visits: baseline and Day 150.

The ACDP^®^ application (for iOS/Android) and *Adhera Digital Companion Platform* collected user engagement data, psychometric responses, and wearable metrics. Participants were provided with *Fitbit Inspire 3* devices (Fitbit Inc., a Google company; San Francisco, CA, USA) to record physical activity, sleep, and heart rate data.

### 2.7. Data Collection and Outcome Measures

Data were collected at baseline and at 5 months; however, a second phase of the intervention will be collected at month 10 and month 12 (follow-up).

At the mid-point of the intervention (5 months), the following domains were assessed:

#### 2.7.1. Primary Outcomes (Clinical and Physical Outcomes)

- Anthropometric data: Weight, height, BMI, and waist circumference.
- Body composition: Body fat % and muscle mass % via bioimpedance.

#### 2.7.2. Secondary Outcomes

##### Engagement Outcomes

- Level of engagement: data were reported by the informatics team based on the following indicators of interaction within the digital application: messages read, questionnaires completed, access to educational units, quizzes performed, and messages sent through the chat.

Engagement scores were calculated by adding the total number of messages to read, ratings given to messages, questionnaires answered, quizzes answered, and educational units accessed. For classification of level of engagement, we split all users into three groups based on where their score falls relative to the data distribution:

(1) Low users with a score ≤ 25th percentile (bottom 25%)
(2) Medium users between 25th and 75th percentile
(3) High users with a score ≥ 75th percentile (top 25%)

##### Usability Outcomes

- Usability: assessed through the System Usability Scale (SUS) [10] to evaluate the perceived usability of the ACDP^®^.

Responses are converted into a standardized score ranging from 0 to 100, where higher values indicate better usability. Scores above 68 are generally interpreted as above average, while scores above 80 indicate excellent usability.

### 2.8. Ethical Considerations

The clinical trial was conducted in accordance with the principles of the Declaration of Helsinki and Good Clinical Practice (GCP) guidelines. The study protocol was reviewed and approved by the Research Ethics Committee of Miguel Servet Children’s University Hospital (Zaragoza, Spain) where the study was carried out. Written informed consent was obtained from all participating caregivers and legal guardians prior to study enrollment. The investigators and clinicians from Miguel Servet Hospital were responsible for data collection, while Adhera Health oversaw monitoring, data consolidation, and analysis. All authors had full access to the study data, participated in the preparation of the manuscript, approved the final version for publication, and ensured the accuracy and integrity of the data as well as the fidelity of the study to its original protocol.

### 2.9. Statistical Analysis

All statistical analyses were performed using R (version 4.5.1; 2025-06-13) in RStudio (version 2025.09.1+401). The analytic sample consisted of 40 participants with complete clinical and physical outcome data at baseline (Day 1) and follow-up (Day 150). Descriptive statistics were used to characterize participants and to summarize all clinical variables at both time points. Pre–post differences (Δ = Day 150 − Day 1) in BMI, weight, body fat percentage, muscle mass percentage, and waist circumference were analyzed using paired *t*-tests. This approach was selected to evaluate within-participant changes and to quantify the magnitude of clinical improvements observed during the intervention period. Usability measures (System Usability Scale, SUS) were available for a subset of participants (*n* = 31), as some participants did not complete the electronic questionnaire. To examine whether perceived usability was associated with clinical improvements, correlations between SUS total scores and clinical change variables were computed using Spearman’s rank correlation coefficient (ρ), given the non-normal distribution of several outcome variables and the ordinal nature of usability scores.

Engagement with digital intervention was captured through three ordered categories (Low, Medium, High). Because engagement represents an ordinal construct, and clinical change variables did not consistently meet assumptions of normality or linearity, the association between engagement level and clinical outcomes was evaluated using Spearman’s ρ. To further explore potential linear trends, supplementary analyses were conducted using Pearson’s correlation coefficient (r), treating engagement as a quasi-continuous variable (1 = Low, 2 = Medium, 3 = High). This analysis leverages the full numeric ordering of categories, increases statistical power under approximate linearity, and permits assessment of effect direction and magnitude on an interval scale. Correlations were also examined within each engagement subgroup to explore possible differential behavioral or clinical patterns. All statistical tests were two-tailed, and a *p*-value < 0.05 was considered statistically significant.

Given the exploratory and pilot nature of the study, no adjustments were applied for multiple comparisons; therefore, correlation analyses should be interpreted as hypothesis-generating rather than confirmatory. Data management, preprocessing, and visualization were conducted using the tidyverse (version 4.5.1), stats (bundled with R 4.5.1), and readxl packages (version 1.4.5) within the R environment (R Foundation for Statistical Computing, Vienna, Austria). As these analyses correspond to the interim assessment at Day 150 of a 300-day intervention, findings are interpreted as preliminary.

## 3. Results

From March to October 2025, a total of 40 participating families were recruited while already undergoing pharmacological treatment with semaglutide and expressing willingness to participate in a combined intervention. Two families withdrew during the initial months of the study and were replaced by two additional families to maintain the target sample size of 40. Up to Day 150, all participants have remained in the study. Clinical assessments were conducted at baseline, at 1 month, at 2 months, and at 5 months, with the latter corresponding to the mid-intervention time point of the trial.

The mean age of participants (children and adolescents) was 14.05 years. Most were female and born in Spain, and the majority reported adhering to diet and exercise recommendations. Demographic characteristics are summarized in Table 1.

### 3.1. Clinical/Physical Outcomes

Clinical and physical outcomes were assessed between the baseline visit (Day 1) and the mid-intervention follow-up (Day 150). Significant pre–post improvements were observed in all evaluated variables, as detailed in Table 2.

The range of weight change in the sample extended from a 0.7 kg increase to a maximum reduction of 21.9 kg, whereas changes in body mass index ranged from a 0.2 kg/m^2^ increase to a maximum decrease of 8.66 kg/m^2^. Paired *t*-tests showed significant improvements across all clinical outcomes from Day 1 to Day 150. Participants experienced a mean reduction of 4.51 kg/m^2^ in BMI (SD = 2.12; *t* = 13.48; *p* < 0.001), a mean decrease of 1.21 in BMI SDS (SD = 0.71; *t* = 13.48; *p* < 0.001), and a mean reduction of 1.20 in Weight SDS (SD = 0.57; *t* = 7.73; *p* < 0.001), along with an average weight loss of 11.42 kg (SD = 5.95; *t* = 12.13; *p* < 0.001). Body fat percentage decreased by 5.63 points (SD = 4.61; *t* = 7.73; *p* < 0.001), while muscle mass percentage increased by 4.47 points (SD = 4.18; *t* = −6.75; *p* < 0.001). Waist circumference was reduced by an average of 8.69 cm (SD = 6.61; *t* = 8.31; *p* < 0.001). Together, the direction and magnitude of these changes suggest consistent improvements in body composition and metabolic risk markers over the 150-day period.

### 3.2. Associations Between Clinical Changes, Digital Engagement, and Usability

To explore whether participant engagement with the digital intervention and perceived usability were related to clinical improvements, correlation analyses were conducted using both Spearman’s ρ and Pearson’s r. Engagement level, treated as an ordinal construct, demonstrated modest positive associations with changes in several clinical and physical outcomes, although these did not reach statistical significance. Usability scores, assessed through the System Usability Scale (SUS), showed similarly weak-to-moderate correlations with clinical change variables in the subset of participants with available data. The SUS evaluation resulted in a score of 72, reflecting usability that is above the normative average and aligning with a good usability classification on established SUS interpretation scales. These exploratory analyses provide preliminary evidence of potential trends linking digital interaction with treatment response, as detailed in Table 3, Table 4 and Table 5.

Table 3 examines whether higher usability (measured by the System Usability Scale, SUS) is associated with greater clinical improvements from Day 1 to Day 150. No significant correlations were found between usability (SUS score) and changes in BMI, weight, body fat percentage, muscle mass, or waist circumference (all *p* > 0.35). These results suggest that perceived usability of the digital intervention did not meaningfully influence the degree of clinical improvement in this sample (*n* = 31).

No significant correlations were found between engagement level and changes in BMI, weight, body fat percentage, muscle mass percentage, or waist circumference (all *p*-values > 0.35). These findings indicate that engagement level did not show a detectable relationship with the magnitude of clinical change in this pilot study.

Given the ordinal nature of the engagement variable, exploratory associations with changes in clinical outcomes were initially examined using Spearman’s ρ to assess potential monotonic relationships without imposing distributional assumptions (Table 4). As a pilot and observational analysis, these analyses were conducted to explore potential patterns, not to establish conclusive associations. As a result, non-parametric rank correlations may be insufficiently sensitive to detect subtle linear dose–response patterns across engagement categories, particularly when engagement may reflect an underlying continuous behavioral construct. The resulting correlation estimates, shown in Table 5, therefore provide a complementary perspective to the non-parametric analysis and an exploratory perspective on engagement–outcome relationships, without implying causal or confirmatory inference.

### 3.3. Exploratory Engagement-Level Interpretation

#### 3.3.1. Low Engagement

Correlation coefficients ranged from 0.289 to 0.400, indicating weak to moderate positive trends; however, none reached statistical significance (all *p* > 0.25). Changes in BMI, weight, and body fat percentage showed small positive associations, while muscle mass and waist circumference exhibited negligible correlations.

Conclusion: No evidence of an association between low engagement and clinical changes was observed; findings are exploratory and hypothesis-generating only.

#### 3.3.2. Medium Engagement

Correlations in the medium-engagement group were generally weak and centered around zero (r between −0.312 and 0.0888; all *p* > 0.20). The largest trend was a non-significant negative association for body fat percentage (r = −0.312).

Conclusion: No clear relationship between medium engagement and clinical outcomes.

#### 3.3.3. High Engagement

The high-engagement group showed slightly stronger—but still non-significant—associations. The change in body fat percentage presented the largest coefficient (r = 0.509, *p* = 0.091), suggesting a possible moderate trend that did not reach significance. Other clinical variables showed weak correlations (r < 0.18, all *p* > 0.58).

Conclusion: A potential trend was observed for body fat percentage, but no statistically meaningful associations emerged. However, these findings should be interpreted cautiously and considered hypothesis-generating only.

Table 6 presents summary statistics for wearable-derived data collected from 19 pediatric participants using Fitbit Inspire 3 devices. Mean values and measures of dispersion are reported for all variables, including calories burned, steps per day, daily activity time, heart rate, and sleep duration. Data were obtained in a real-world setting, and daily synchronization of the devices was not consistent across participants, which led to variability in the completeness of the datasets and should be taken into account when interpreting these results.

To complement the primary statistical analyses, a series of boxplots were generated to visually characterize the distribution of clinical and physical outcomes at Day 1 and Day 150, as well as the magnitude of individual change across the intervention period. These visualizations provide additional insight into variability, central tendencies, and potential outliers that may not be fully captured by summary statistics alone. Furthermore, to illustrate potential behavioral patterns related to adherence, supplementary boxplots stratified by engagement level depict differences in clinical change scores across High, Medium, and Low engagement groups. Together, these graphical representations offer a descriptive overview of participant trajectories and support the interpretation of the quantitative findings presented in the main text (Supplementary Materials: Figures S1 and S2).

## 4. Discussion

In this combined clinical trial of pharmacotherapy and a digital intervention targeting families of children with obesity, we observed that, in our cohort, weight decreased by an average of 11.42 kg (SD 5.95), indicating a more homogeneous treatment response compared to previously reported semaglutide studies in youth (e.g., −7.03 ± 7.50 kg) [8]. Approximately two thirds of participants lost between 5.5 kg and 17.4 kg, reflecting substantial and clinically meaningful reductions in body weight throughout the 150-day intervention period. Similarly, BMI decreased by an average of 4.51 points (SD 2.12; *p* < 0.001), a change that is consistent with previously reported effects of semaglutide in adolescents, where mean BMI reductions around −6.0 (95% CI −7.3 to −4.6) have been documented [9]. In a 12-month follow-up study, a mean reduction of −6.48% in body fat percentage was reported among youth receiving GLP-1-based therapy combined with lifestyle support [11].

Semaglutide demonstrated superior outcomes compared with other pharmacological options. It exhibited greater effects in reducing weight, BMI, and BMI z-score, [12] and appeared to be the most effective and safest option among the four GLP-1 receptor agonists evaluated in children and adolescents with obesity or overweight [12,13]. A 2024 systematic review and meta-analysis in adolescents without diabetes reported that semaglutide produced significantly larger reductions in body weight and BMI compared with other GLP-1 receptor agonists [14]. Similarly, a 2025 network meta-analysis encompassing thirty randomized controlled trials and more than 3800 youths found that semaglutide outperformed the majority of available anti-obesity medications; key outcomes included weight, BMI, and waist circumference [15]. Overall, the consistency and magnitude of these results underscore semaglutide as a leading pharmacologic option for managing obesity in children and adolescents.

Interventions that combine semaglutide with lifestyle-focused treatment showed reductions in BMI with waist circumference [7,16] % weight [16,17] and % body fat [11] while muscle mass increased. However, these last two clinical variables have received considerably less attention in interventions combining pharmacotherapy with traditional and digital lifestyle-support tools. Our 5-month interim assessment demonstrated a similar reduction in body fat percentage (−5.63%), indicating that clinically relevant improvements in adiposity can emerge relatively early during combined pharmacological and lifestyle treatment. These findings fall within this therapeutic range while reflecting the shorter follow-up period and real-world conditions of the present study.

Although the cohort showed substantial clinical improvement over the 150-day period, we did not observe significant associations between caregiver engagement, perceived usability, and changes in BMI, weight, or body composition. Several factors may account for this. The overall effect of the combined intervention was large and relatively uniform, which may have limited the detection of additional benefits related to higher digital engagement. In addition, the sample size—especially for usability assessments (*n* = 31)—reduced the statistical power for correlation analyses and subgroup comparisons. In addition, the engagement metric, derived from aggregated digital activity logs, may not fully capture the household-level behavioral changes that influence obesity outcomes.

Nevertheless, the moderate association between higher engagement and greater reductions in body fat percentage among highly engaged caregivers points to a potential emerging pattern warranting further evaluation. It is plausible that sustained and more intensive use of the digital program could lead to more pronounced improvements in adiposity as family routines become established, and the pharmacological effect stabilizes. Analysis of the full 300-day dataset will be necessary to determine whether these early indications reflect meaningful long-term differences.

In our study, the average sleep duration reported by participants was comparable to the values observed by George et al. [18] in adolescents monitored with commercially available wrist-worn devices. However, both the mean sleep duration and the average number of daily steps were lower than those reported in a similar adolescent cohort by Zhang et al. [19], with sleep being nearly one hour shorter and more than 2000 fewer daily steps. These differences may reflect variations in participant characteristics, daily routines, or adherence to wearable device use across studies. In our sample, the average daily energy expenditure estimated by the wearable devices was 2423 kcal/day; this value is broadly consistent with expected energy requirements for children and adolescents with moderate levels of daily activity, especially considering the mean age of the sample (14.05 years), although individual needs vary according to age, body composition, and growth stage. Prior research has shown that daily energy expenditure in youth commonly ranges between approximately 2000 and 3000 kcal/day depending on activity level and developmental stage [20].

Taken together, these findings offer meaningful implications for the management of pediatric obesity alongside GLP-1 therapy. The pronounced reductions in weight and improvements in body composition highlight the short-term effectiveness of pharmacologic treatment in specialized pediatric endocrinology settings. Complementing this approach with a caregiver-focused digital program extends support into the home environment, where daily lifestyle decisions shape adherence and behavioral change.

The study’s strengths are noteworthy. Conducted in a real-world public hospital context, it enrolled adolescents already receiving pharmacologic therapy and followed them as part of a structured 10-to-12-month protocol, with the present analysis focusing on the first 150 days. The integration of GLP-1 treatment with a caregiver-centered digital intervention reflects a contemporary, family-oriented approach to obesity management. Standardized assessments of BMI, weight, waist circumference, and bioimpedance-based body composition during routine care improve the external validity of the findings. Additionally, engagement and usability were captured through established metrics, offering a clear view of how families interacted with the digital platform.

However, several limitations should be considered. This study was designed as an observational, single-arm pre–post study, which inherently limits causal inference regarding the effects of the digital program. The absence of a control group restricts attribution of observed changes to the intervention, while the small sample size and single-center setting reduce generalizability and limit the detection of modest associations. The requirement that families adopt a mobile application may have favored the participation of more motivated or digitally proficient caregivers. Moreover, the current analysis is restricted to Day-150 data, leaving long-term outcomes—such as sustained adherence and weight maintenance—uncertain. Lastly, app-derived engagement indicators may not fully reflect the quality of interaction or the degree to which digital recommendations translated into concrete behavioral changes; qualitative elements, such as caregiver follow-up or interactions with the health coach (an optional feature provided by the ACDP), could be explored in future work to complement this level of engagement.

## 5. Conclusions

In this DHI for pediatric obesity, children and adolescents receiving GLP-1-based pharmacologic therapy in combination with the Adhera^®^ Caring Digital Program showed improvements in key clinical outcomes over the first 150 days of follow-up. Observed reductions in BMI, body weight, body fat percentage, and waist circumference, together with increases in muscle mass, reflect favorable changes in body composition and central adiposity within a real-world public hospital setting. These observed changes are broadly consistent with—and in some cases numerically greater than—previously reported outcomes for GLP-1 receptor agonists in similar pediatric populations.

Despite these favorable clinical changes, we did not observe statistically significant associations between caregiver engagement or perceived usability of the digital program and short-term clinical outcomes. A moderate, non-significant correlation between higher engagement and reductions in body fat percentage was observed; this represents an exploratory signal only and should be interpreted as hypothesis-generating rather than indicative of a dose–response relationship which requires longer follow-up and larger samples to be fully characterized. These findings underscore the need to refine how digital engagement is measured and to capture the behavioral changes occurring more effectively within families.

This study demonstrates the feasibility of integrating a caregiver-centered digital support tool into routine pediatric endocrinology care alongside GLP-1 pharmacotherapy. Such tools may help extend clinical guidance into the home environment, where day-to-day lifestyle decisions occur, and could support more scalable models of family-based obesity management. The upcoming assessments at Day 300 and Day 360 will be essential to determine the durability of the observed clinical benefits, clarify the contribution of digital engagement over time, and inform future controlled trials aimed at optimizing digital support strategies within pediatric obesity treatment pathways.

## Figures and Tables

**Table 1:** Demographic and clinical characteristics of the study population.

Variable	*n* (%)/Mean ± SD
Age (years)	14.05 ± 1.04
Gender	
Male	15 (37.5%)
Female	25 (62.5%)
Country of birth	
Spain	34 (85%)
Venezuela	2 (5%)
Honduras	2 (5%)
Peru	1 (2.5%)
Romania	1 (2.5%)
Diet and exercise	
Yes	36 (90%)
No	4 (10%)

Abbreviations: SD, standard deviation; *n*, number of participants.

**Table 2:** Paired *t*-test results for clinical and physical outcome changes from Day 1 to Day 150.

Clinical/Physical Outcomes	Mean Day 1	Mean Day 150	Mean Change	Δ SD	*t*	*p* Value
BMI (kg/m^2^)	35.35	30.84	−4.51	2.12	13.481	<0.001 *
BMI SDS	3.96	2.75	−1.21	0.71	10.68	<0.001 *
Weight (kg)	95.37	83.95	−11.42	5.95	12.132	<0.001 *
Weight SDS	3.60	2.40	−1.2	0.57	13.11	<0.001 *
Bioimpedance (% body fat)	42.69	37.06	−5.63	4.61	7.727	<0.001 *
Bioimpedance (% muscle mass)	55.28	59.75	4.47	4.18	−6.752	<0.001 *
Waist circumference (cm)	108.8	100.11	−8.69	6.61	8.307	<0.001 *
Waist circumference SDS	6.716	5.35	−1.366	1.29	6.597	<0.001 *

Abbreviations: SDS, standard deviation score; Δ SD, standard deviation of the change; Bioimpedance, bioelectrical impedance analysis; *t*, Student’s *t* statistic; * statistically significant (*p* < 0.0005).

**Table 3:** Correlation results for Clinical/Physical Outcomes Differences and SUS Scores.

Clinical/Physical Outcomes	Spearman Rho (ρ)	*p* Value	*n*
Δ BMI	0.1725	0.3534	31
Δ BMI SDS	0.0899	0.6365	31
Δ Weight (kg)	0.0969	0.6041	31
Δ Weight SDS	0.1343	0.4793	31
Δ Bioimpedance (% body fat)	0.0532	0.7763	31
Δ Bioimpedance (% muscle mass)	−0.0997	0.5936	31
Δ Waist circumference (cm)	0.1326	0.4769	31

Abbreviations: Δ, change from baseline to Day 150.

**Table 4:** Correlation Results for Clinical/Physical Outcomes Differences and Engagement Level.

Clinical/Physical Outcomes	Spearman Rho (ρ)	*p* Value	*n*
Δ BMI	−0.0977	0.5485	40
Δ BMI SDS	0.1301	0.4298	40
Δ Weight (kg)	−0.0818	0.6157	40
Δ Weight SDS	0.0582	0.7247	40
Δ Bioimpedance (% body fat)	0.1515	0.3506	40
Δ Bioimpedance (% muscle mass)	−0.0820	0.6149	40
Δ Waist circumference (cm)	0.1148	0.4807	40

**Table 5:** Correlation Results for Clinical/Physical Outcomes Differences and Individual Engagement Level (Low, Medium, High).

	Low Engagement *n* = 10		Medium Engagement *n* = 18		High Engagement *n* = 12	
Clinical/Physical Outcomes	r	*p* Value	r	*p* Value	r	*p* Value
Δ BMI (kg/m^2^)	0.289	0.4180	0.0888	0.7290	0.177	0.5812
Δ Weight (kg)	0.339	0.3376	0.061	0.8096	0.166	0.6068
Δ Bioimpedance (% body fat)	0.400	0.2518	−0.312	0.2080	0.509	0.0910
Δ Bioimpedance (% muscle mass)	−0.014	0.9696	−0.142	0.5737	0.468	0.1253
Δ Waist circumference (cm)	0.058	0.8734	−0.029	0.9102	0.160	0.6191

**Table 6:** Descriptive Statistics of Daily Activity, Heart Rate, and Sleep Duration.

Variable	*n*	Mean	SD	Minimum	Q1	Median	Q3	Maximum
Calories burned (kcal/day)	19	2423.59	728.78	1220.54	1960.53	2373.75	3001.55	3643.15
Steps per day	19	6617.66	4007.94	0	4611.19	7107.47	8933.08	15,158.28
Activity (hours/day)	16	4.16	1.21	1.21	3.81	4.31	4.81	6.57
Heart rate (bpm)	16	74.53	6.11	64.09	70.27	74.70	78.51	85.06
Sleep duration (hours)	16	6.91	1.19	4.70	6.48	6.94	7.47	9.29

Abbreviations: Q1, first quartile; Q3, third quartile; kcal, kilocalories; bpm, beats per minute.

## Data Availability

The datasets used and analyzed during the current study are available from the corresponding author upon reasonable request.

## Supplementary Materials

The following supporting information can be downloaded at: https://www.mdpi.com/article/10.3390/jcm15010222/s1, Figure S1: Clinical and Physical Outcomes at Baseline (Day 1) and Mid-Intervention (Day 150). Figure S2: Clinical Change Scores (Δ) Stratified by Engagement Level.

## Abbreviations

The following abbreviations are used in this manuscript:

ACDP	Adhera^®^ Caring Digital Program
AI	Artificial Intelligence
AEMPS	Agencia Española de Medicamentos y Productos Sanitarios
BMI	Body Mass Index
CI	Confidence Interval
DHI	Digital Health Intervention
EMA	European Medicines Agency
GCP	Good Clinical Practice
GLP-1	Glucagon-Like Peptide 1
mHealth	Mobile Health
SUS	System Usability Scale
SD	Standard Deviation
iOS	iPhone Operating System
R	R Statistical Software
