# Supplementary material for: Clinical Outcomes Associated with the Use of a Family-Based Digital Support Program in Patients with Pharmacologic Treatment for Obesity
Source: J Clin Med. 2025 Dec 27;15(1):222. doi: 10.3390/jcm15010222 (PMC12786419; doi:10.3390/jcm15010222)
Supplement: Supplementary file 1 [file jcm-15-00222-s001.zip › jcm-4053294-Supplementary.pdf]

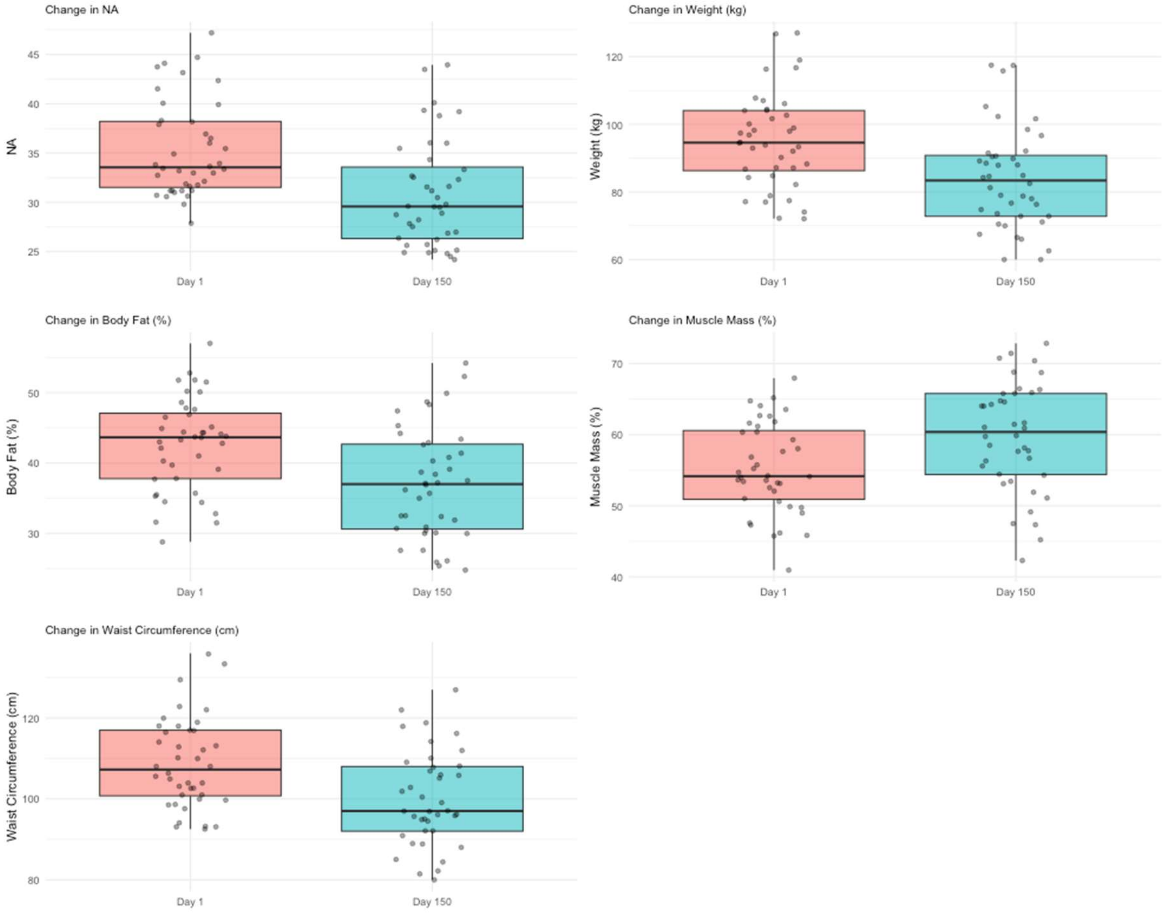

Figure S1. Clinical and Physical Outcomes at Baseline (Day 1) and Mid-Intervention (Day 150).

Figure S1. Distribution of clinical and physical outcome measures at baseline (Day 1) and at the mid-intervention follow-up (Day 150). Boxplots display values for BMI, body weight, body fat percentage, muscle mass percentage, and waist circumference across both time points. Individual data points are shown to illustrate variability and participant-level trajectories. These visualizations complement the paired t-test results reported in the main text and highlight overall improvements in adiposity and body composition during the first 150 days of the intervention.

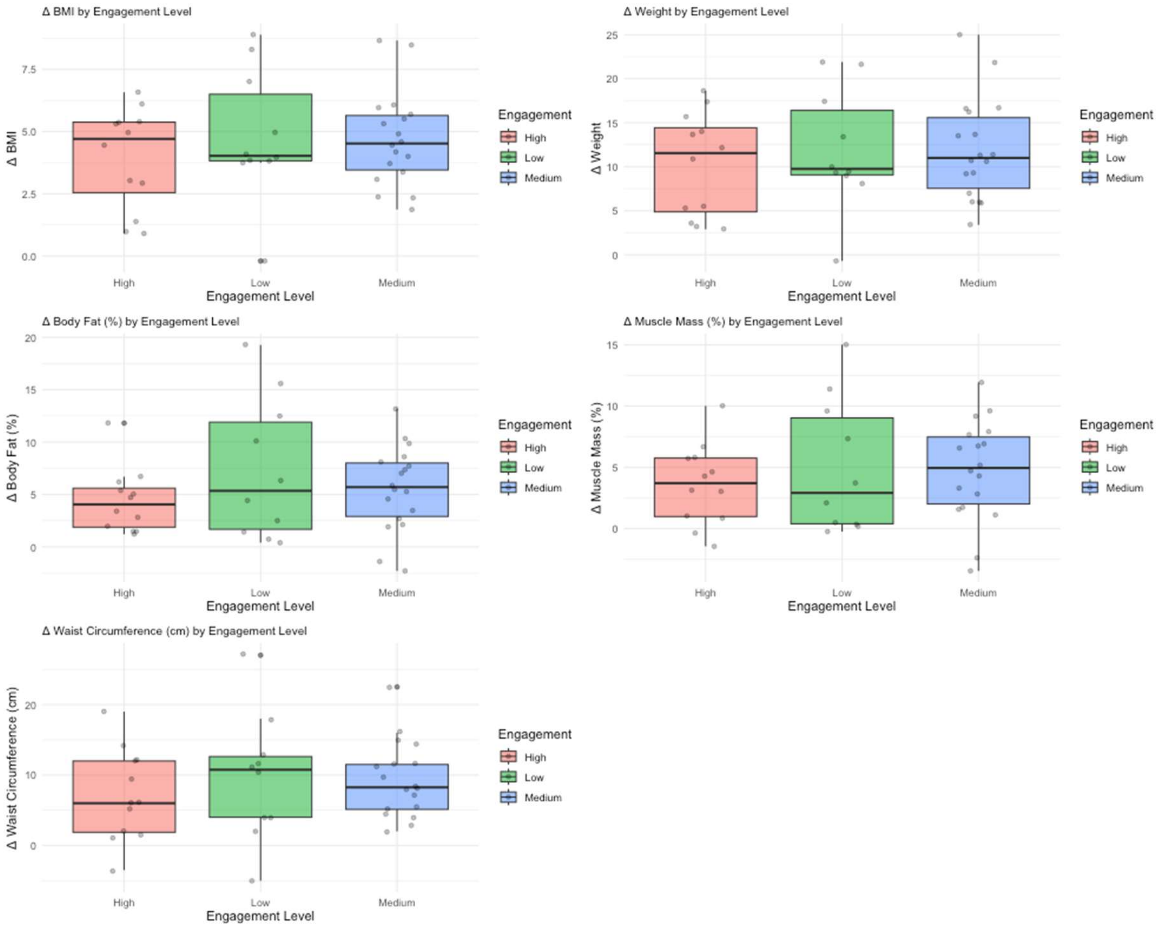

Figure S2. Clinical Change Scores (Δ) Stratified by Engagement Level.

Figure S2. Boxplots illustrating clinical change scores (Δ = Day 150 − Day 1) for BMI, weight, body fat percentage, muscle mass percentage, and waist circumference stratified by engagement level (High, Medium, Low). Each panel shows the distribution and variability within engagement categories, along with individual data points to visualize participant-level dispersion. These graphical representations complement the correlation analyses presented in Tables 4 and 5, offering visual insight into potential patterns between digital engagement and clinical response.
